# Integrated Metabolomic and Biological Profiling of *Cymodocea nodosa*: Tissue-Specific Chemical Diversity and Fouling-Relevant Activity

**DOI:** 10.3390/biology15211909

**Published:** 2026-10-22

**Authors:** Bianca Gabbrielli, Matteo Oliva, Giorgia Fumagalli, Donatella Degl’Innocenti, Marzia Vasarri, Alessio Lenzi, Carlo Pretti

**Affiliations:** 1 Department of Veterinary Sciences, University of Pisa, Viale delle Piagge 2, 56124 Pisa, Italy; bianca.gabbrielli@phd.unipi.it (B.G.); alessio.lenzi@unipi.it (A.L.); 2 Interuniversity Consortium of Marine Biology and Applied Ecology “G. Bacci” (CIBM), Viale N. Sauro 4, 57128 Livorno, Italy; oliva@cibm.it (M.O.); fumagalli@cibm.it (G.F.); 3 Department of Experimental and Clinical Biomedical Sciences, University of Florence, Viale Morgagni 50, 50134 Florence, Italy; donatella.deglinnocenti@unifi.it (D.D.); marzia.vasarri@unifi.it (M.V.)

**Keywords:** *Cymodocea nodosa*, marine natural products, metabolomic profiling, bioactivity, marine biofouling

## Abstract

Seagrasses are important components of coastal ecosystems and represent a source of chemically diverse natural products with potentially relevant biological activities. This study aimed to characterize the biochemical and metabolomic profiles of leaves and rhizomes of the Mediterranean seagrass *Cymodocea nodosa* and to compare the biological activity of extracts from the two tissues across different marine organisms. Hydroalcoholic extracts were characterized by biochemical assays and untargeted liquid chromatography–high-resolution mass spectrometry, while biological responses were assessed using *Aliivibrio fischeri*, *Phaeodactylum tricornutum*, *Dunaliella tertiolecta*, *Acartia tonsa*, and the fouling serpulid *Ficopomatus enigmaticus*. Leaves and rhizomes differed in their biochemical characteristics but shared most putatively annotated metabolites, including phenolic acids, flavonoids, lipid-derived metabolites, and amino acid derivatives. Semi-quantitative analysis revealed tissue-associated differences in relative LC-HRMS signal intensities. Biological responses were species- and endpoint-dependent, with *F. enigmaticus* showing the highest sensitivity, particularly to the rhizome extract (EC50 = 48.8 mg/L), whereas the other organisms generally responded at higher concentrations. Overall, these findings highlight tissue-associated chemical and biological differences in *C. nodosa* extracts and identify rhizomes as particularly interesting for further investigation into the relationships between seagrass metabolites and fouling-related biological responses.

## Simple Summary

Marine organisms produce a wide variety of natural substances that can influence their interactions with other organisms and may provide useful compounds for environmentally sustainable applications. In this study, we investigated the Mediterranean seagrass *Cymodocea nodosa*, comparing leaves and rhizomes to determine whether their chemical composition is associated with different biological activities. Chemical analyses revealed that the two tissues shared most of the identified substances, although differences in their relative representation were observed. Their biological effects were evaluated using several marine organisms representing bacteria, microalgae, a small crustacean, and a tube-building marine worm. The biological responses differed considerably among organisms and between the two plant tissues. The strongest response was observed in the fouling worm *Ficopomatus enigmaticus*, particularly with the extract from underground stems, whereas the other organisms were generally less sensitive. These findings highlight differences in the chemical characteristics and biological activity of *Cymodocea nodosa* extracts and identify rhizomes as particularly promising for further investigation. The results provide a basis for further research into their ecological significance and their potential use in developing more sustainable approaches to marine biofouling.

## 1. Introduction

Marine biofouling, the undesirable colonization of submerged surfaces by microorganisms, algae and invertebrates, remains a major challenge for maritime industries and coastal infrastructure. Fouling increases hydrodynamic drag on ship hulls, leading to higher fuel consumption, greenhouse gas emissions and operational costs, while also compromising the performance of aquaculture facilities, offshore platforms and desalination systems. In addition, biofouled vessels facilitate the spread of non-indigenous species, representing an important pathway for marine biological invasions [1,2]. Consequently, the development of environmentally sustainable antifouling technologies has become a global priority [3].

For decades, marine biofouling has been controlled using coatings containing broad-spectrum biocides. Although tributyltin (TBT)-based paints were highly effective, their severe ecological impacts resulted in a worldwide ban by the International Maritime Organization [4]. Copper-based coatings supplemented with booster biocides subsequently became the dominant antifouling strategy; however, increasing evidence has demonstrated that many of these compounds adversely affect non-target marine organisms [3,5]. These concerns have stimulated the search for environmentally compatible alternatives based on natural products capable of preventing surface colonization through selective ecological mechanisms rather than indiscriminate toxicity [6,7,8].

Marine organisms constitute an extraordinary source of chemically diverse secondary metabolites that mediate ecological interactions such as competition, defence, allelopathy and resistance to microbial colonization [9,10]. Marine natural products have therefore attracted considerable attention as potential antifouling agents because many interfere with bacterial communication, biofilm formation or larval settlement while exhibiting lower environmental persistence than conventional biocides [7,11]. Recent studies further suggest that the ecological functions of these metabolites often arise from chemically complex mixtures acting additively or synergistically rather than from individual compounds acting alone [10,12].

Among marine primary producers, seagrasses represent an attractive yet still underexplored source of bioactive natural products [13]. Living permanently submerged, these marine angiosperms are continuously exposed to bacteria, epiphytic algae and sessile invertebrates, creating strong selective pressure for the evolution of sophisticated chemical defence systems [14,15]. Their metabolomes comprise phenolic compounds, flavonoids, fatty acids, glycolipids and numerous other specialized metabolites involved in antimicrobial defence, protection against oxidative stress, and regulation of plant–microbe interactions [16]. Beyond their ecological importance as ecosystem engineers, seagrasses are increasingly recognized as promising reservoirs of compounds with pharmaceutical and biotechnological potential [17].

The Mediterranean seagrass *Cymodocea nodosa* is widely distributed throughout the Mediterranean Sea and adjacent eastern Atlantic coasts, where it forms highly productive meadows that provide essential ecosystem services, including carbon sequestration, sediment stabilization and nursery habitats for numerous marine organisms [14,15]. Previous phytochemical investigations have identified phenolic acids, flavonoids, fatty acids, sterols and other metabolites exhibiting antioxidant, antimicrobial and cytotoxic activities, suggesting that *C. nodosa* possesses a rich chemical repertoire [18,19]. Nevertheless, compared with the extensively investigated *Posidonia oceanica*, the metabolome of *C. nodosa* remains comparatively poorly characterized, and the ecological significance of its chemical diversity is still largely unresolved [16].

Recent developments in high-resolution mass spectrometry have considerably expanded the capacity to investigate plant metabolomes. Untargeted liquid chromatography–high-resolution mass spectrometry (LC-HRMS) enables the simultaneous detection of hundreds of metabolites, providing comprehensive metabolic fingerprints that better represent the chemical complexity of natural extracts than traditional targeted phytochemical approaches [20,21]. However, although metabolomic studies have increasingly been applied to marine plants, they have rarely been integrated with ecotoxicological investigations aimed at identifying the metabolite classes potentially responsible for antifouling activity.

Similarly, most studies investigating the antifouling properties of seagrasses have focused either on chemical characterization or biological activity, whereas investigations combining comprehensive metabolomic profiling with multitrophic ecotoxicological assessment remain scarce [22,23]. Moreover, tissue-specific chemical specialization has received limited attention despite the contrasting ecological roles of leaves and rhizomes, which experience different environmental pressures and are therefore expected to allocate defensive metabolites differently [24].

To address these knowledge gaps, the present study integrates colorimetric biochemical characterization, untargeted LC-HRMS metabolomics, and standardized marine assays to investigate the antifouling potential of hydroalcoholic extracts obtained from leaves and rhizomes of *C. nodosa*. Specifically, (i) total polyphenol content, antioxidant capacity and polysaccharide content were determined; (ii) the metabolomic profiles for leaves and rhizomes were qualitatively and semi-quantitatively characterized; (iii) biological activity was evaluated using model organisms representative of different stages of marine biofouling, including bacteria, microalgae, crustaceans and macrofouling invertebrates; and (iv) the relationship between tissue-specific metabolomic composition and toxic effects of the extracts was explored. The study was not designed to identify a purified antifouling principle or to demonstrate coating performance. Rather, it tests whether crude *C. nodosa* extracts contain fouling-relevant biological activity sufficient to justify subsequent bioassay-guided fractionation, targeted compound confirmation and application-specific validation.

## 2. Materials and Methods

### 2.1. Cymodocea nodosa Extract Preparation

*Cymodocea nodosa* ((Ucria) Asch., 1870) plagiotropic rhizomes with their leaves were collected along the coast of Livorno (Italy) in July 2025. Only detached bundles of *C. nodosa* uprooted by anchoring activities were collected. Separate extracts were prepared from leaves and rhizomes.

The collected leaves and rhizomes of *C. nodosa* were carefully washed with distilled water to remove surface-associated epiphytes and salts and were subsequently allowed to air-dry at 37 °C. Once dried, the plant material was finely cut into small, relatively homogeneous fragments using scissors. For each extraction, 4 g of dried plant material were weighed. The material was reduced to small fragments to increase the surface area exposed to the extraction solvent and facilitate a more reproducible extraction process. Extraction was performed with 70% (*v*/*v*) ethanol for 24 h at 37 °C. Subsequently, samples were centrifuged at 4000 rpm for 15 min at 25 °C. The supernatant was collected and centrifuged again (8000 rpm for leaf tissue and 10,000 rpm for rhizome tissue) for 15–20 min at 25 °C to remove residual debris. The final supernatant was aliquoted (1 mL aliquots) in 1.5 mL tubes. Finally, the ethanol extracts were dried at 37 °C for two days. The dry extracts were stored at −20 °C until use. At the time of each experiment, the extracts were reconstituted in 0.5 mL of DMSO (Merck KGaA, Darmstadt, Germany) to obtain stock solutions at final concentrations of 40 mg/mL for leaf extracts and 100 mg/mL for rhizome extracts. Aliquots of these stock solutions were subsequently diluted as required for the different analytical and bioassay procedures. All tests involving DMSO were conducted with a 1% DMSO control [25]. For the high-resolution mass spectrometry analysis, dried extracts were instead reconstituted in 1 mL of a 1:1 (*v*/*v*) H_2_O:ACN mixture, as described in Section 2.3.

### 2.2. Water Media Preparation

Natural seawater was collected in Quercianella (Livorno, Italy). Measured parameters were 8.11 pH and 39 psu salinity. Before use, the seawater was filtered at 0.45 μm. Salinity was adjusted to 30 psu with ultrapure water. The seawater used for all experiments was referred to as filtered natural seawater (FNSW). Artificial seawater (ASW) was prepared following ASTM standards [26] and kept in 25 L HDPE tanks at 5° C.

### 2.3. Chemical Characterization of Extracts

#### 2.3.1. Colorimetric Biochemical Characterization of Extracts

Colorimetric assays were performed to characterize selected biochemical constituents of the extracts and to evaluate their antioxidant properties. The characterization included the quantification of total polyphenols content (TPC) and total carbohydrate content (TCC), expressed as gallic acid equivalents (GAE) and glucose equivalents (GE), respectively, as well as the assessment of antioxidant capacity and radical-scavenging activity using the FRAP and DPPH assays, respectively. The latter assays provide measures of the functional antioxidant properties of the extracts and do not represent quantitative measurements of specific antioxidant metabolites.

Total Polyphenol Content (TPC) in *C. nodosa* extracts was determined using the Folin-Ciocalteau colorimetric method [27]. Briefly, scalar volumes of *C. nodosa* extracts (final volume: 20 µL) were added to the wells of a 96-well microplate, followed by the addition of 100 µL Folin–Ciocalteu’s phenol reagent (diluted 1:10 in distilled water). After 5 min of incubation at 25 °C, 80 µL of 7.5% sodium carbonate solution was added to each well. The reaction mixture was incubated for 2 h at 25 °C. The absorbance was measured at 595 nm using a microplate reader. Gallic acid (0.5 mg/mL) was used to generate a calibration curve in the range of 0–10 µg, and TP values were expressed as milligrams of gallic acid equivalent (GAE) per milliliter of *C. nodosa* extracts.

Total Carbohydrate Content (TCC) of *C. nodosa* extracts was quantified using the phenol-sulfuric acid colorimetric method [27]. Equal volumes of *C. nodosa* extracts were mixed with 5% phenol and concentrated sulfuric acid in a 96-well microplate. After thorough mixing and incubation at 90 °C for 5 min, the absorbance was recorded at 490 nm. D-glucose (0.5 mg/mL) was used as a standard in the range of 0–10 µg to construct the calibration curve. Carbohydrate content was expressed as milligrams of D-glucose equivalent (GE) per milliliter of *C. nodosa* extracts.

The antioxidant capacity (FRAP assay) of *C. nodosa* extracts was assessed using the Ferric Reducing Antioxidant Power (FRAP) assay [27]. In this study, distilled water was added to *C. nodosa* extract samples to a final volume of 50 µL per well, followed by the addition of 200 µL of Ferrozine™ reagent (Sigma-Aldrich, Merck KGaA, Darmstadt, Germany; P9762), prepared as 10 mM Ferrozine™ in 40 mM HCl, 20 mM ferric chloride, and 0.03 M acetate buffer at pH 3.6 in a 1:1:10 ratio. The reaction was incubated at 25 °C, and the absorbance was measured at 595 nm. Ascorbic acid (0.1 mg/mL) was used as the reference standard, and a calibration curve was constructed in the range of 0–4 µg. Antioxidant capacity was reported as milligrams of ascorbic acid equivalent (AAE) per milliliter of *C. nodosa* extracts.

The Radical Scavenging Activity (DPPH assay) of *C. nodosa* extracts was evaluated using the DPPH (Sigma-Aldrich, Merck KGaA, Darmstadt, Germany; D9132), a method based on the reduction in the stable radical DPPH to a non-radical form upon interaction with antioxidants [27]. Scalar volumes of *C. nodosa* extracts were diluted with methanol (final volume: 100 µL) in a 96-well plate and mixed with 100 µL of freshly prepared DPPH solution (0.25 mg/mL in methanol). The mixture was incubated in the dark at 25 °C for 30 min, after which absorbance was measured at 490 nm. Ascorbic acid (0.5 mg/mL) was used as the reference compound in the range of 0–4 µg, and results were expressed as milligrams of ascorbic acid equivalent (AAE) per milliliter of *C. nodosa* extracts.

For all chemical characterization assays, standard curves were generated by plotting absorbance values against known concentrations of the respective standards: gallic acid for TP, D-glucose for TC, and ascorbic acid for both FRAP and DPPH assays. The concentrations of the analytes in *C. nodosa* extracts were calculated using linear regression analysis. Each experiment was performed in triplicate, and the results were expressed as mean ± standard deviation (SD) to ensure reproducibility and statistical reliability.

#### 2.3.2. Untargeted LC-HRMS Characterization of Extracts: Procedures and Data Processing

##### Chemicals and Equipment

Acetonitrile (ACN) and water were purchased from Carlo Erba (Milan, Italy) at UHPLC-MS grade. Formic acid (FA, for LC-MS LiChropur™, purity 98–100%) was purchased from Merck Millipore (Milan, Italy). Ammonium acetate for LC-MS (AmAc), at a purity of >99%, was purchased from AnalytiChem (Zedelgem, Belgium). Color-fixed pH test strips (0.0–14.0) were purchased from Carlo Erba (Milan, Italy). A VELP Scientifica ZX3 Advanced Vortex Mixer (Usmate, Italy), a Multi Reax 115V shaker (Heidolph Scientific, Germany), and a Heraeus Megafuge 8R centrifuge (Thermo Scientific, USA) were used for sample mixing and centrifugation. Before LC injection, each sample or standard solution was stored in the autosampler in glass vials purchased from Agilent Technologies (USA).

##### High-Resolution Mass Spectrometry Analysis

Dried seagrass extracts were reconstituted with 1 mL of a 1:1 *v*/*v* H_2_O:ACN mixture and vortexed for 1 min and centrifuged at 5000× *g* for 10 min at 25 °C. An aliquot was then transferred to an HPLC vial for analysis. Instrumental analysis was performed using a 1290 Infinity II LC system coupled to a 6546 quadrupole-time-of-flight mass spectrometer (LC-Q/TOF), equipped with a Dual Jet Stream electrospray (Dual AJS ESI) ionization source (Agilent Technologies, USA). The separation was achieved at 35 °C using an Infinity Lab Poroshell 120 SB-C18 column (2.1 mm I.D. × 150 mm length, 2.7 μm particle size) coupled with a Poroshell 120 SB-C18 (2.1 × 5 mm length, 2.7 μm) guard column. The LC mobile phase consisted of water (A) and acetonitrile (B), both containing formic acid at 0.1% *v*/*v* in positive polarity, and 5 mM AmAc (pH 6) (A) and 95:5 *v*/*v* ACN:H_2_O + 5 mM AmAc (B) in negative polarity. The composition of the mobile phase changed over time as follows: 2% (B) for 3 min, 2–35% (B) in 10 min, 35–100% (B) in 7.0 min, isocratic for 3.0 min, re-equilibration to initial conditions (2% B) in 0.5 min, isocratic up to 30 min. A volume of 2 µL was injected for each sample, and the autosampler was kept at 4 °C. The mass spectrometer operated in positive ESI mode using the following parameters: capillary voltage 3500 V, nebulizer gas 35 psi, drying gas flow rate 10 L/min, drying gas temperature 325 °C, sheath gas temperature 350 °C, and sheath gas flow rate 11 L/min. Nozzle and fragmentor voltages were set at 0 and 150 V, respectively, while skimmer voltage was 65 V, and OCT 1 RF Vpp voltage was 750 V. For negative ESI mode, the same parameters were employed except for nozzle voltage (500 V). The MS and MS/MS scan rates were 2 spectra/s and 3 spectra/s, respectively. Full scan data were acquired over an *m*/*z* range of 100–1200 in both ionization modes. MS/MS spectra were obtained at collision energies of 10, 20, and 40 V over an *m*/*z* range of 40–1000 in negative mode and 40–750 *m*/*z* in positive mode. The quadrupole isolation width was set at 1.3 *m*/*z*. Instrument control and data acquisition were performed using MassHunter Workstation software B.10.1. All data were acquired in both profile and centroid modes in the extended dynamic range (10 GHz). Calibration and tuning of the instrument were performed according to the manufacturer’s recommended procedures. Mass accuracy of the acquired ions was ensured by performing continuous internal calibration during analysis, using reference solution signals at *m*/*z* 121.0509 and 922.0098 in positive mode, and *m*/*z* 119.0363 and 1033.9881 in negative mode [28].

##### HRMS Data Processing, Feature Identification, and Annotation

Raw data acquired from LC-qTOF in .d format were pre-processed using Agilent MassHunter Profinder software (version 10.0). Untargeted feature extraction was performed using a Batch Recursive Feature Extraction algorithm, by grouping technical injection replicates (*n* = 2) for each sample to enable retention time and mass alignment across the sample batch. Additionally, Molecular Feature Extraction (MFE) was applied to remove background noise and detect molecular features in the total ion chromatograms, searching for compound isotope distributions. The parameters used for feature extraction are reported in Table 1. Features showing a coefficient of variation (CV) < 30% in peak area across two replicates of the same sample were further selected for compound identification and annotation. Putative identification was performed by comparing experimental mass spectral data with reference spectra available in the METLIN database or in public databases, including LIPIDMAPS, Human Metabolome Database (HMDB), and Mass Bank of North America (MoNA). Feature annotation was carried out by searching for positive ions as [M + H]+, [M + Na]+, and [M + NH_4_]+ adducts, and negative ions as [M − H]− and [M + CH3]−. Metabolites with an overall identification score > 80 and a mass accuracy within ±5 ppm were selected for MS/MS analysis and putative annotation. Approximately 100 candidate compounds were further investigated by examining MS/MS spectra in Agilent MassHunter Qualitative Analysis software (version 10.0) acquired at collision energies of 10, 20, and 40 V. The parameters used for the identification included a forward score > 50 and a reverse score > 80, comparing fragmentation patterns against those in reference databases. Identification confidence levels were assigned according to the Metabolomics Standards Initiative (MSI) guidelines [20]. Level 2 was assigned when MS/MS spectral matching with database reference spectra was possible, and level 3 when MS/MS confirmation was not available in such databases. TableS S1 and S2 report the compounds putatively annotated within this study for *C. nodosa* leaves and rhizomes, respectively.

To further investigate tissue-associated differences in metabolite profiles, a semi-quantitative comparison of LC-HRMS peak areas was performed between leaf and rhizome extracts of *C. nodosa*. Features reported in Tables S1 and S2 were matched based on identical putative annotations and ion adducts, with a maximum retention-time difference of 0.03 min and a mass difference within 10 ppm. For each matched feature, mean peak areas obtained from two technical injections were used to calculate the fold change between tissues. Fold changes were expressed as the ratio between the higher and lower peak areas, with the corresponding tissue identified as showing the higher relative signal intensity. A two-fold threshold was adopted as a descriptive criterion to highlight the most pronounced differences between tissues. Features detected in only one tissue were reported separately. The resulting comparison is presented in Table S4. Because the analysis was based on technical injection replicates and did not involve absolute metabolite quantification, the observed differences were interpreted as relative variations in LC-HRMS signal intensity without assigning statistical significance.

### 2.4. Bioassay-Based Characterization

#### 2.4.1. *Aliivibrio fischeri* Inhibition of Bioluminescence

Gram-negative bacterium *Aliivibrio fischeri* was purchased as freeze-dried bacterial cells (strain no. 19A4002A, Ecotox LDS, Pregnana Milanese, Milan, Italy). The test for acute luminescent bacteria followed the standardized method [29]. The principle of this method is the evaluation of *A. fischeri* bioluminescence reduction after 30 min of exposure to aqueous substances with respect to a negative control. ASW was used for both sample preparation and dilution and as a negative control for the assay. The assay was performed in duplicate for each sample/concentration. Light emission was measured using the Microtox^®^ Model 500 analyzer and results were analyzed with the MicrotoxOmni Software (v. 4.2), developed by Modern Water (UK). Zinc sulphate heptahydrate acted as the reference toxicant (Table S3).

#### 2.4.2. *Dunaliella tertiolecta* and *Phaeodactylum tricornutum* Growth Inhibition

The green alga *Dunaliella tertiolecta* and the diatom *Phaeodactylum tricornutum* strains were originally purchased from the Culture Collection of Algae and Protozoa (CAAP) at the Scottish Marine Institute and clones were maintained under laboratory conditions. Tests were conducted by the use of standardized methods for *D. tertiolecta* [26] and for *P. tricornutum* [30].

Algal cultures were inoculated 72 h before the beginning of the assay in order to obtain a suspension in the exponential growth phase. After that pre-culture phase, algal concentrations were estimated spectrophotometrically (λ = 670 nm); the suspensions were diluted with growth medium to obtain a specific concentration of 10^6^ cells/mL.

Aliquots of 20 µL were added to 2 mL control/sample/dilution replicates (*n* = 3), previously prepared in 24-well plates. Growth medium acted as a control and was also used for sample dilution preparation.

Plates were incubated for 72 h at a temperature of 20 ± 1° C under continuous illumination (6000–8000 lux). After this period, algal concentration in each well was measured spectrophotometrically (λ = 670 nm). Potassium dichromate acted as reference toxicant for both assays (Table S3).

#### 2.4.3. *Acartia tonsa* Naupliar Immobilization

The calanoid copepod *Acartia tonsa* stock was purchased by Guernsey Sea Farms at Parc Lane in Vale, Guernsey. The culture was established in 8 L glass tanks and maintained under specific conditions: filtered seawater with a 0.45 µm filter, salinity 30 ± 2, pH 8.2 ± 0.1, temperature 20 ± 1° C, and a 14 h light and 10 h darkness photoperiod. Cultures were fed twice weekly with a mix of *Tetraselmis suecica* and *Isochrysis galbana*. For ecotoxicological valuation, the acute toxicity test was performed, involving hatched nauplii over a 24 to 96 h test period [31]. The 5 nauplii were placed in each well of 24-well plates, 2 wells per replicate, with each well filled with 2.5 mL of samples. Each sample or dilution was set up in triplicate. The plates were kept at a temperature of 20 ± 1° C, with a photoperiod of 14 h of light and 10 h of darkness. The number of mobile nauplii was recorded every 24 h from the start of the test. Ni^2+^ acted as the reference toxicant (Table S3).

#### 2.4.4. *Ficopomatus enigmaticus* Larval Development

*Ficopomatus enigmaticus* reef portions were sampled in the regional park of S. Rossore-Migliarino (Pisa, Italy). These reefs were kept in laboratory tanks under specific conditions: pH 8.2 ± 0.1, temperature 22 ± 1° C, oxygen saturation > 90%, 14 h light and 10 h darkness photoperiod, and salinity 30 ± 2° C [32]. Organisms were fed daily with an *Isochrysis galbana* suspension (about 10^5^ cells/mL).

To collect gametes, calcareous tubes of *F. enigmaticus* were gently broken using tweezers to remove adult worms. The worms were then placed individually in the wells of a 24-well plate containing 1 mL of FNSW, after being quickly washed in tap water. Tube breaking induced the release of gametes within 10 min [32]. Female individuals were identified, and eggs were collected in a beaker filled with 300 mL of FNSW. Broken and immature eggs were removed by rinsing the suspension three times with fresh FNSW. Egg concentration in the rinsed suspension was adjusted to 300 cells/mL with FNSW. After egg collection, spermatozoa were collected from 3 to 5 males, mixed and an aliquot was inoculated into the egg suspension to allow fertilization. The fertilization process was monitored in the first hour, then 1 mL of fertilized egg suspension was inoculated into each 10 mL sample/dilution replicate (three replicates per sample/dilution/control). All samples were incubated at 20 ± 1° C for 48 h. Then 1 mL of 37% formaldehyde was added to each tube to stop development. The test tubes were then stored at 5 ± 2° C until microscope analysis [33]. The percentage of inadequately developed larvae was then calculated. Cu^2+^ acted as the reference toxicant (Table S3).

### 2.5. Statistical Analysis

The EC_10_ and EC_20_ values were calculated for each ecotoxicological bioassay, based on the specific test conditions. Growth inhibition in *P. tricornutum* and *D. tertiolecta* was analyzed using the INTERPOLATION v2.0 software [34]. Bioluminescence inhibition in *A. fischeri* was assessed with MicrotoxOmni^®^, which automatically calculates the ecotoxicological parameters. For the assays on larval development in *F. enigmaticus* and bioluminescence inhibition in *A. tonsa*, the 48 h survival values were determined using the MOSAIC web-interface for statistical analyses in ecotoxicology [35]. Statistical analysis of the mean effects at each concentration was conducted using ANOVA, followed by Tukey’s post-test for multiple comparisons, implemented in GraphPad Prism^®^ version 5.0. Differences in mean effects at each concentration were also compared to the control using Dunnett’s post-test, which was performed with the same software across all bioassays.

## 3. Results

### 3.1. Colorimetric Biochemical Characterization of Extracts

Colorimetric analyses demonstrated clear biochemical differences between hydroalcoholic extracts obtained from leaves and rhizomes of *C. nodosa* (Table 2). The two tissues were characterized in terms of total polyphenol content (TPC), total carbohydrate content (TCC), and antioxidant properties assessed by FRAP and DPPH assays. Rhizome extracts consistently exhibited higher values for all measured parameters than leaf extracts, indicating a greater overall content of extractable bioactive compounds.

TCC was substantially higher in rhizome extracts than in leaf extracts. Rhizome extracts also exhibited greater antioxidant capacity in both the FRAP and DPPH assays. Likewise, the TPC was approximately two-fold higher in rhizomes than in leaves. Overall, the colorimetric characterization revealed tissue-specific differences in both the measured biochemical constituents and the antioxidant properties of the extracts.

These observations indicate that, under the extraction conditions employed in the present study, rhizomes contained higher levels of TCC and TPC and exhibited greater antioxidant activity than leaves. However, the FRAP and DPPH results represent functional antioxidant properties of the extracts and cannot be directly interpreted as quantitative measures of specific antioxidant metabolites.

### 3.2. Untargeted LC-HRMS Characterization of Extracts

Untargeted LC-HRMS analysis enabled the putative annotation of a chemically diverse metabolome in both leaf and rhizome extracts of *C. nodosa* (Tables S1 and S2). Metabolite annotation was performed using accurate mass measurements, isotope distribution, MS/MS fragmentation patterns, and database matching, allowing the assignment of compounds according to the Metabolomics Standards Initiative (MSI) criteria.

Overall, both tissues exhibited a complex metabolomic composition comprising representatives of primary and specialized metabolism. The detected compounds included organic acids, amino acids and amino acid derivatives, carbohydrates, quaternary ammonium compounds, hydroxycinnamic acid derivatives, flavonoids, hydroxyquinone-related metabolites, glycolipids, sulfoquinovosyl diacylglycerols (SQDGs), saturated and unsaturated fatty acids, and several nitrogen-containing metabolites.

Comparison of the LC-HRMS datasets revealed a substantial overlap in the putatively annotated metabolites detected in leaf and rhizome extracts of *C. nodosa*. Of the 55 annotated features, 54 were detected in both tissues, whereas only one feature, assigned to a hydroxycinnamate derivative isomer, was detected exclusively in rhizomes.

Despite this extensive qualitative overlap, semi-quantitative comparison of LC-HRMS peak areas revealed tissue-associated differences in the relative signal intensities of several shared metabolites. Using a two-fold change as a descriptive threshold, 14 features showed differences between tissues, with 11 displaying higher signals in leaves and three in rhizomes (Table S4).

Leaf-enriched features included two isorhamnetin diglycoside isomers, trigonelline, proline betaine, azelaic acid, benzoic acid, a pentitol isomer, two oxidized fatty acid derivatives, and two sulfoquinovosyl diacylglycerols (SQDGs). Conversely, a phenolic acid diglucoside derivative, citric acid, and sucrose showed higher relative signals in rhizomes. Among these, the phenolic acid diglucoside derivative exhibited the largest difference, with an approximately 13.8-fold higher signal in rhizomes.

These results indicate that tissue-associated chemical differentiation in *C. nodosa* is primarily reflected in differences in the relative LC-HRMS signal intensities of shared metabolites rather than in the occurrence of distinct tissue-exclusive metabolite repertoires.

### 3.3. Bioassay-Based Screening

The biological activity of leaf and rhizome extracts was evaluated using a battery of standardized marine bioassays encompassing organisms representative of successive stages of the biofouling process, from bacterial colonizers to macrofouling invertebrates (Table 3).

Reference toxicants were included in each standardized assay as performance controls, with their EC50 values and confidence limits reported in Table S3. These reference substances were not treated as direct potency equivalents of the crude extracts, because extract concentrations are expressed as total dry mixture mass rather than as concentrations of defined active ingredients. Both extracts produced measurable but strongly species- and endpoint-dependent responses. The clearest fouling-relevant effect occurred in *F. enigmaticus*, whereas several EC50 values for the other species were above the highest tested concentration (180 mg/L). Accordingly, the results are interpreted as preliminary evidence of fouling-relevant biological activity rather than as a demonstration of broad-spectrum antifouling efficacy.

#### 3.3.1. *Aliivibrio fischeri* Bioluminescence Inhibition: Bacterial Metabolic Activity

**Figure 1:**
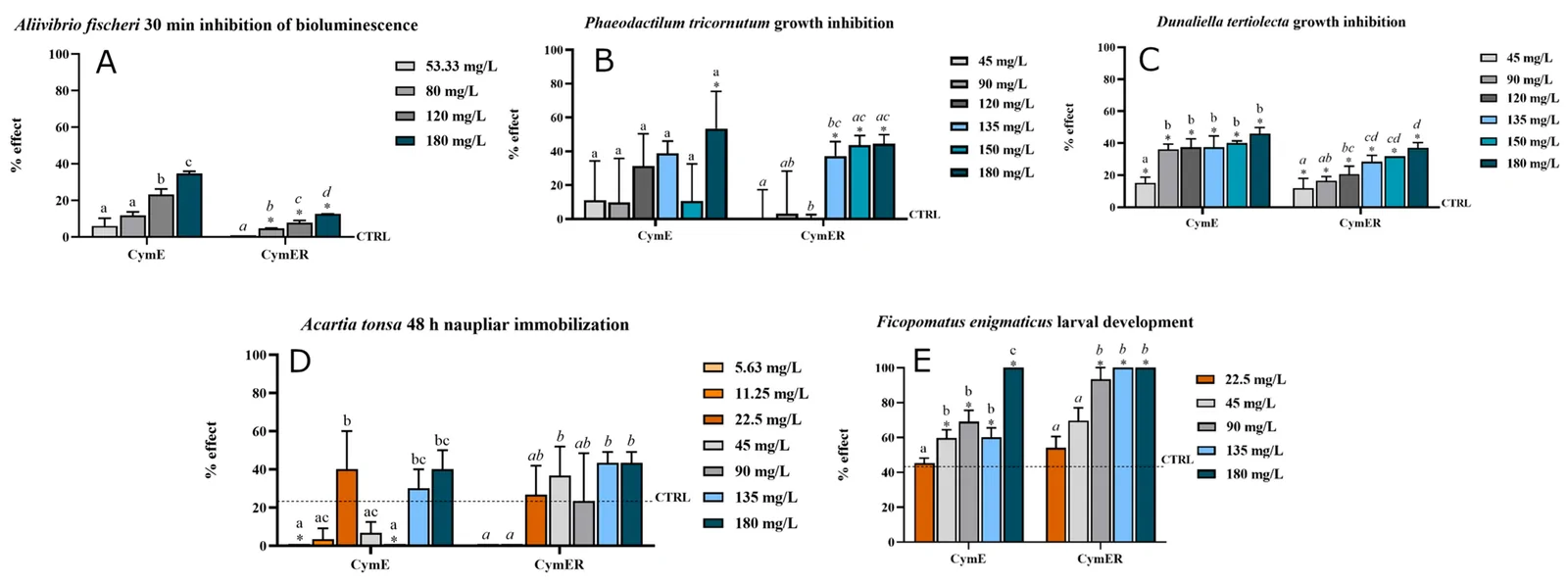
Ecotoxicological tests on (**A**) *A. fischeri*; (**B**) *P. tricornutum*; (**C**) *D. tertiolecta*; (**D**) *A. tonsa*; and (**E**) *F. enigmaticus*, tested with leaf extracts (CymE) and rhizome extracts (CymER) of *C. nodosa*. The dashed line indicates the average effect observed in the control samples. An ANOVA was conducted across all concentrations for each extract, followed by Tukey’s post hoc test to identify significant differences between concentrations, with different letters denoting statistical significance (*p* < 0.05). Additionally, Dunnett’s post hoc test was used to compare each sample concentration to the control, with * indicating statistically significant differences (*p* < 0.05).

*A. fischeri* responded to both extracts, although the response was limited at the concentration range tested. For the leaf extract, EC10 and EC20 values were 84.77 and 124.9 mg/L, respectively, while EC50 was >180 mg/L (Figure 1). For the rhizome extract, EC10 was 166.6 mg/L and both EC20 and EC50 were >180 mg/L. Because *A. fischeri* is a standardized ecotoxicological model rather than a representative surface-colonizing bacterium, these data indicate interference with bacterial metabolic activity.

#### 3.3.2. *D. tertiolecta* and *P. tricornutum* Growth Inhibition: Microalgal Endpoints

The two marine microalgae showed comparatively low sensitivity (Figure 1). For *P. tricornutum* exposed to leaf extract, EC10 was 152.29 mg/L, while EC20 and EC50 were >180 mg/L; all effect concentrations for the rhizome extract were >180 mg/L. For *D. tertiolecta*, EC10, EC20 and EC50 values were >180 mg/L for both extracts. These results indicate that the extracts were not broadly algicidal within the tested range and should not be interpreted as direct evidence of inhibition of algal surface colonization.

#### 3.3.3. *Acartia tonsa* Naupliar Immobilization: Non-Target Crustacean Endpoint

*A. tonsa* was included principally as a non-target planktonic organism to provide an initial indication of ecological selectivity (Figure 1). The leaf extract produced EC10 and EC20 values of 133 and 156 mg/L, respectively, with EC50 > 180 mg/L. The rhizome extract was more active, with EC10 and EC20 values of 33.9 and 72.1 mg/L, respectively, while EC50 remained > 180 mg/L. These responses are therefore relevant to preliminary toxicity effects toward crustaceans.

#### 3.3.4. *Ficopomatus enigmaticus* Larval Development: Fouling-Relevant Macroinvertebrate Endpoint

The strongest biological response was observed in *F. enigmaticus* (Figure 1). For the leaf extract, EC10, EC20 and EC50 values were 80.6, 85.1 and 92.5 mg/L, respectively. The rhizome extract showed greater activity, with corresponding values of 27.5, 34.0 and 48.8 mg/L. Because *F. enigmaticus* is a serpulid polychaete that contributes to hard fouling, this developmental endpoint is the most directly fouling-relevant response in the present screening. Taken together, the bioassays show that *C. nodosa* extracts are biologically active, but the magnitude and ecological meaning of the responses differ among endpoints. The dataset therefore supports prioritization of the active extracts and endpoints for subsequent fractionation and settlement-specific testing.

#### 3.3.5. Integrating Metabolomic and Ecotoxicological Datasets

Considered together, the LC-HRMS and bioassay datasets provide complementary insights into the chemical diversity and biological activity of *C. nodosa* extracts. Leaves and rhizomes displayed partially overlapping but distinct metabolite profiles, accompanied by species- and endpoint-dependent biological responses. Among the putatively annotated metabolites, hydroxycinnamic acids, flavonoids, glycolipids, fatty acids and nitrogen-containing compounds represent chemical classes with reported biological properties potentially relevant to interactions with marine organisms. The occurrence of these chemically and functionally diverse metabolites may contribute to the broad spectrum of responses observed across the bioassay battery and supports the interest of *C. nodosa* as a source of bioactive marine natural products. Selected metabolites and their previously reported biological activities are summarized in Table 4, providing a basis for future targeted analyses and bioassay-guided fractionation aimed at identifying the constituents most closely associated with the observed effects.

## 4. Discussion

### 4.1. Chemical Diversity and Ecological Significance of the Cymodocea nodosa Metabolome

The integration of biochemical characterization, untargeted LC-HRMS profiling and multitrophic biological screening provides a comprehensive picture of the chemical diversity and bioactivity of *C. nodosa*. Leaves and rhizomes displayed distinct biochemical properties and metabolomic fingerprints, indicating a marked tissue-specific organization of the seagrass metabolome. This differentiation is consistent with the contrasting physiological and ecological functions of above- and belowground tissues and supports the growing evidence that seagrasses represent a rich source of structurally and functionally diverse marine natural products [16,24]. Leaves constitute the photosynthetically active interface with the water column and are continuously exposed to irradiation, oxidative stress, herbivory and epiphytic colonization. Rhizomes, in contrast, are embedded in sediments and contribute to anchorage, resource storage and interactions with sediment-associated microorganisms. These contrasting environments can favour different patterns of metabolic investment, particularly in compounds involved in protection, signalling and biotic interactions [16,24]. The higher total polyphenol content and antioxidant capacity observed in rhizome extracts are consistent with such tissue-specific specialization [42] and suggest substantial investment in protective chemistry in belowground tissues. In addition, the antioxidant capacity could play a key role in antifouling activity as indicated by some authors [43], who introduced the antioxidant N-acetylcysteine (NAC) on a functionalized polyvinyl chloride surface in order to scavenge Reactive Oxygen Species (ROS). By doing so, it effectively disrupts the oxidative cross-linking mechanisms that marine mussels rely on to firmly adhere to underwater surfaces, providing a non-toxic method to prevent biofouling [43]. However, our findings should not be interpreted as direct evidence that polyphenols alone determine the activity of the rhizome extracts. Total polyphenol content and antioxidant assays represent broad biochemical indicators and do not identify the compounds responsible for a given biological effect.

LC-HRMS profiling revealed a broad spectrum of putatively annotated metabolites, including phenolic acids, flavonoids, fatty acids, glycolipids, amino acid derivatives, and other specialized metabolites. Although the untargeted approach was primarily qualitative, the comparison of LC-HRMS peak areas provided additional semi-quantitative information on tissue-associated metabolic patterns. Notably, leaves and rhizomes shared nearly all annotated features, indicating substantial qualitative overlap in their chemical composition. Nevertheless, several metabolites exhibited pronounced differences in relative signal intensity between tissues (Table S4), suggesting that chemical differentiation in *Cymodocea nodosa* is primarily associated with the relative representation of shared metabolites rather than the occurrence of tissue-exclusive compounds. These differences should be interpreted cautiously, as LC-HRMS peak areas do not provide absolute metabolite concentrations, and the analysis was based on technical rather than independent biological replicates. Targeted analyses using authentic standards will therefore be necessary to confirm and quantify selected compounds [20,21].

Hydroxycinnamic acid derivatives represented a substantial group among the annotated phenolic metabolites, consistent with previous phytochemical investigations of *C. nodosa* and other *Cymodocea* species [18,19,22,23]. Chicoric acid, in particular, has been reported as a characteristic component of *C. nodosa* and is associated with several biological properties, including antioxidant activity [19]. Interestingly, the semi-quantitative comparison revealed that a phenolic acid diglucoside derivative exhibited a markedly higher LC-HRMS signal in rhizomes, whereas two isorhamnetin diglycoside isomers showed higher signals in leaves (Table S4). This pattern suggests that tissue-associated differences within the phenolic fraction are compound-dependent rather than reflecting a uniform enrichment of phenolic metabolites in either tissue. The higher relative signals of isorhamnetin derivatives in leaves are consistent with the protective functions generally attributed to flavonoids, particularly their involvement in photoprotection and responses to oxidative stress. Flavonoids and related phenolics may also influence microbial membranes, cellular metabolism, and signalling processes [37,44], making this chemical fraction particularly relevant for subsequent activity-guided investigations.

Additional tissue-associated differences were observed among lipid-derived metabolites. In particular, two features putatively annotated as sulfoquinovosyl diacylglycerols (SQDGs) showed higher relative signals in leaves (Table S4). These sulfolipids are characteristic components of photosynthetic membranes and contribute to thylakoid organization and function [45,46]. Their higher relative signals in leaf extracts are therefore consistent with the photosynthetic specialization of aboveground tissues. Similarly, oxo-octadecatrienoic and hydroxy-octadienoic acid derivatives showed higher signals in leaves. These compounds belong to the broader group of oxidized fatty acid derivatives, including oxylipins, which participate in plant stress responses, redox regulation, and defence-related signalling [47,48]. Azelaic acid, also showing a higher relative signal in leaves, has been implicated in systemic defence priming in terrestrial plants [48]. Although comparable functions have not been demonstrated for these specific metabolites in *C. nodosa*, their distribution may reflect differences in lipid-associated metabolism between photosynthetic and belowground tissues [49]. More generally, fatty acids and glycolipids may interact with cellular membranes and membrane-associated processes, potentially contributing to biological effects alongside phenolic constituents [50].

Other metabolites showing higher relative signals in leaves included trigonelline, proline betaine, and a pentitol isomer. These compounds belong to chemical groups associated with stress-related metabolism and, in some plant systems, osmotic adjustment. Their distribution may therefore be consistent with the physiological demands of leaf tissues exposed to fluctuating environmental conditions, although their specific roles in *C. nodosa* remain unknown. Conversely, sucrose and citric acid showed higher relative signals in rhizomes, potentially reflecting differences in carbon allocation and primary metabolism between aboveground and belowground tissues. Recent metabolomic investigations of seagrasses have highlighted the responsiveness of metabolite profiles to environmental conditions, further supporting the importance of considering metabolic composition within a physiological and ecological context [51]. Together, these observations suggest that tissue-associated chemical patterns may reflect the contrasting physiological roles of leaves and rhizomes rather than the presence of fundamentally different metabolite repertoires.

The relationship between chemical composition and biological activity should nevertheless be considered at the extract level. Natural-product mixtures may produce additive, synergistic, or antagonistic interactions, and their overall biological properties cannot always be predicted from individual constituents [52]. Interestingly, although most metabolites exhibiting at least two-fold differences showed higher LC-HRMS signals in leaves, rhizome extracts produced the strongest response in the fouling serpulid *Ficopomatus enigmaticus*. This observation highlights that biological activity cannot be inferred simply from the number of relatively enriched metabolites and may instead depend on the identity, potency, and interactions of specific constituents. The chemical diversity of *C. nodosa* extracts, together with their biological effects across different marine organisms, therefore provides a rationale for subsequent bioassay-guided fractionation to identify the constituents contributing most strongly to the observed responses.

These findings may also have ecological significance. Seagrasses are long-lived organisms continuously exposed to microorganisms, epiphytes, herbivores, and sessile organisms, and their specialized metabolites may contribute to several ecological functions, including oxidative protection, regulation of microbial interactions, and defence against surface colonization [16]. The coexistence of shared metabolites with tissue-associated differences in relative signal intensity may therefore reflect different physiological requirements and ecological interactions in leaves and rhizomes [42]. Previous investigations of marine plant extracts, including those obtained from other *Cymodocea* species such as *C. serrulata*, have also reported biologically active constituents and effects on microalgae, crustaceans, and bacteria [53]. Although direct comparisons are limited by differences in extraction procedures and experimental conditions, these studies further support the interest in seagrass-derived natural products as potential sources of biologically active compounds.

### 4.2. Fouling-Relevant Biological Activity, Limitations and Next Steps

The multitrophic bioassay battery revealed a clear species- and endpoint-dependent biological profile for *C. nodosa* extracts. This is particularly relevant in an antifouling context because marine surface colonization involves interactions among microorganisms, microalgae and invertebrate larvae rather than a single biological target. Biofilms can modify surface properties and generate chemical cues that influence subsequent colonization and larval settlement [54,55,56,57]. Assessing different biological groups therefore provides a broader perspective on the activity spectrum of the extracts.

The response of *A. fischeri* indicates that components of the extracts can interfere with bacterial metabolic activity. Microorganisms represent an important component of marine surface colonization, and natural products capable of influencing bacterial growth, adhesion or cell-to-cell signalling have attracted considerable attention in antifouling research [58]. The occurrence of phenolic and flavonoid metabolites in *C. nodosa* provides several candidate chemical classes that may contribute to such interactions, as observed also for other species of the genus *Cymodocea* [53]. The two marine microalgae, *P. tricornutum* and *D. tertiolecta*, were comparatively less sensitive, with most calculated effect concentrations exceeding 180 mg/L. This differential response is informative because it demonstrates that the activity of the extracts is not uniform across biological groups. In the context of environmentally compatible antifouling development, such selectivity is potentially valuable, as an ideal antifouling agent should interfere with surface colonization while limiting effects on non-target marine primary producers. Among the organisms tested, the serpulid *F. enigmaticus* showed the strongest fouling-relevant response, particularly following exposure to the rhizome extract, which produced an EC50 of 48.8 mg/L. This finding is noteworthy because serpulid polychaetes are important components of hard macrofouling communities and can form persistent calcareous tubes on submerged artificial surfaces. The greater activity of the rhizome extract towards *F. enigmaticus*, together with its distinctive metabolomic fingerprint and higher polyphenol content and antioxidant capacity, identifies belowground tissues as a particularly interesting source for further activity-guided investigation. Settlement-specific assays will be especially valuable for determining whether this response translates into interference with larval attachment and colonization.

The inclusion of *A. tonsa* complements the fouling-oriented assays by providing information on a non-target planktonic crustacean. The rhizome extract produced greater responses at low-effect levels (EC10 = 33.9 mg/L; EC20 = 72.1 mg/L), indicating that biological selectivity should remain an important criterion during subsequent fractionation. This combination of fouling-relevant and non-target endpoints is useful for identifying fractions that retain activity towards target organisms while displaying a more favourable environmental profile.

Taken together, the biological results reveal an activity spectrum rather than generalized toxicity. The strongest response occurred in the macrofouling-relevant serpulid, whereas the microalgal endpoints were considerably less responsive within the tested concentration range. Such differences among taxa provide useful information for prioritizing both chemical fractions and biological endpoints during subsequent development.

Reference toxicants included in the standardized assays confirmed assay responsiveness and provided useful internal benchmarks (Table S3).

The combined chemical and biological evidence therefore positions *C. nodosa* as a promising source for antifouling-oriented marine natural-product discovery. Bioassay-guided fractionation can now exploit the observed differences between leaves and rhizomes to progressively associate biological responses with specific fractions and chemical constituents. Testing purified compounds alongside reconstructed mixtures and the original extracts will also help determine whether activity is concentrated in individual metabolites or results from interactions among different components of the natural mixture [52].

From an application perspective, incorporation of selected active fractions or compounds into experimental coating systems represents a subsequent stage of particular interest. Coating-leachate assays, settlement experiments and field immersion tests will allow the activity observed under aqueous exposure conditions to be translated into environmentally realistic antifouling scenarios, while simultaneously evaluating release behaviour, persistence and non-target effects. Seasonal and geographical variability in the chemical profile should also be considered when evaluating the reproducibility and potential sourcing of bioactive material, as recent evidence demonstrates pronounced seasonal and site-dependent variation in the volatilome of *C. nodosa* across Mediterranean environments [59].

## 5. Conclusions

The integrated biochemical, metabolomic, and biological characterization of *Cymodocea nodosa* leaf and rhizome extracts revealed substantial qualitative overlap in their chemical composition, accompanied by tissue-associated differences in biochemical characteristics and relative LC-HRMS signal intensities. The annotated metabolites included phenolic acids, flavonoids, lipid-derived compounds, amino acid derivatives, and other specialized metabolites. Biological responses were species- and endpoint-dependent, with the fouling serpulid *F. enigmaticus* showing the highest sensitivity, particularly to the rhizome extract. These findings highlight the biological potential of *C. nodosa* extracts and identify rhizomes as a priority for further investigation of compounds with possible antifouling activity.

Although the present results do not establish a direct relationship between individual metabolites and the observed biological effects, they provide a basis for subsequent bioassay-guided investigations. Future studies should focus on fractionation of the most active extracts, structural confirmation and quantification of candidate metabolites, and settlement-specific assays to distinguish antifouling effects from general toxicity. Evaluation of the most promising fractions or compounds in experimental coating systems, followed by testing under environmentally realistic conditions, will be essential to assess their effectiveness, selectivity, and potential applicability in sustainable marine antifouling strategies.

## Figures and Tables

**Table 1:** Agilent MassHunter Profinder parameters set for Batch Recursive Feature Extraction workflow for both ionization modes.

Parameters		
Peak Filter		10,000 counts
Limit assigned charge states		1–2
Alignment parameters	RT * tolerance	0.50 min
	Mass tolerance	10 ppm
Post-processing filter		Score (MFE **) ≥ 80
Minimum filter matches		100% in at least one sample per group
Minimum find-by-ion RT score		80
Peak integration threshold (height)		10,000

* RT: retention time; ** MFE: molecular feature extraction.

**Table 2:** Biochemical characterization and antioxidant properties of hydroalcoholic extract from leaves and rhizomes of *Cymodocea nodosa*.

Assay	Leaves	Rhizomes
Total Polyphenol Content (TPC), mg GAE/mL	0.7 ± 0.1	1.52 ± 0.23
Antioxidant capacity (FRAP), mg AAE/mL	0.38 ± 0.02	1.21 ± 0.05
Radical-scavenging activity (DPPH), mg AAE/mL	3.6 ± 0.25	8.26 ± 2.9
Total Carbohydrate Content (TCC), mg GE/mL	25.7 ± 2.7	162 ± 15.6

GAE, gallic acid equivalents; AAE, ascorbic acid equivalents; GE, glucose equivalents.

**Table 3:** EC_10_, EC_20_, and EC_50_ values with their respective 95% confidence limits (in square brackets), for *C. nodosa* leaf and rhizome extracts on each bioassayed species.

**Leaves**			
**Species**	**EC_10_**	**EC_20_**	**EC_50_**
*P. tricornutum*	152.29 mg/L [132.2; 233.6]	>180 mg/L	>180 mg/L
*D. tertiolecta*	>180 mg/L	>180 mg/L	>180 mg/L
*A. tonsa*	133 mg/L [122; 144]	156 mg/L [147; 165]	>180 mg/L
*F. enigmaticus*	80.6 mg/L [67.8; 88.1]	85.1 mg/L [76; 89.1]	92.5 mg/L [90.1; 98.4]
*A. fischeri*	84.77 mg/L [70.60;101.8]	124.9 mg/L [105.4;148.0]	>180 mg/L
**Rhizomes**			
**Species**	**EC_10_**	**EC_20_**	**EC_50_**
*P. tricornutum*	>180 mg/L	>180 mg/L	>180 mg/L
*D. tertiolecta*	>180 mg/L	>180 mg/L	>180 mg/L
*A. tonsa*	33.9 [25; 45.9]	72.1 [58.7; 88.6]	>180 mg/L
*F. enigmaticus*	27.5 mg/L [21.3; 34.1]	34 mg/L [27.5; 40.7]	48.8 mg/L [42.8; 55.4]
*A. fischeri*	166.6 mg/L [123.7; 224.5]	>180 mg/L	>180 mg/L

**Table 4:** Ecologically relevant metabolites identified in *C. nodosa* leaf and rhizome extracts and their potential contribution to antifouling activity.

Metabolite	Chemical Class	Reported Biological Activities	Possible Contribution to the Present Study	References
Caffeic acid	Hydroxycinnamic acid derivative	Anti-fouling (algae, bacteria)	May contribute to microbial and algae inhibition	[36]
Chicoric acid	Hydroxycinnamic acid	Antioxidant, antibacterial, antibiofilm	May contribute to bacterial growth inhibition and protection against oxidative stress	[16,19]
Caftaric acid	Hydroxycinnamic acid	Antioxidant, antimicrobial	Possible contribution to inhibition of early microbial colonizers	[16]
Flavonoid derivatives	Flavonoids	Antioxidant, quorum sensing inhibition, antibiofilm	May interfere with bacterial communication and biofilm establishment	[37]
Hydroxyquinone derivatives	Quinones	Redox-active, antimicrobial	Potential interference with microbial respiration and oxidative balance	[10]
Sulfoquinovosyldiacylglycerols (SQDGs)	Glycolipids	Antiviral, antibacterial, membrane-active	Possible alteration of microbial membrane integrity	[38]
Palmitic acid	Saturated fatty acid	Antibacterial, antifouling	May contribute to membrane disruption in microorganisms	[39]
Oleic acid	Unsaturated fatty acid	Antimicrobial, antibiofilm	Possible inhibition of bacterial adhesion	[39]
Linoleic acid derivatives	Polyunsaturated fatty acids	Antimicrobial, signaling	May contribute to ecological defence against epiphytes	[39]
Trigonelline	Quaternary ammonium compound	Osmoprotection, antioxidant, antimicrobial	May increase stress tolerance and indirectly contribute to antimicrobial activity	[40]
Agmatine derivatives	Polyamine derivatives	Stress signalling, antimicrobial	Possible modulation of microbial growth and oxidative stress responses	[41]
Organic acids (citric, malic, succinic acids)	Organic acids	Primary metabolism, metal chelation	Support cellular metabolism and may influence extract acidity and microbial susceptibility	[19]

## Data Availability

Data are contained within the article or Supplementary Materials. Further inquiries can be directed to corresponding author.

## Supplementary Materials

The following supporting information can be downloaded at: https://www.mdpi.com/article/10.3390/biology15211909/s1, Table S1: LC-HRMS analysis of leaves’ extracts of *C. nodosa*; Table S2: LC-HRMS analysis of rhizome’ extracts of *C. nodosa*; Table S3: Evaluation of EC50 values along with associated confidence limits for the selected reference biocides used on each tested species; Table S4: Putatively annotated metabolites showing at least two-fold differences in LC-HRMS peak areas between leaf and rhizome extracts of *C. nodosa*.
