# Supplementary material for: Integrated Metabolomic and Biological Profiling of Cymodocea nodosa: Tissue-Specific Chemical Diversity and Fouling-Relevant Activity
Source: Biology (Basel). 2026 Oct 22;15(21):1909. doi: 10.3390/biology15211909 (PMC13762400; doi:10.3390/biology15211909)
Supplement: Supplementary file 1 [file biology-15-01909-s001.zip › Table S3.pdf]

Tab. S3: Evaluation of EC50 values along with associated confidence limits for the selected reference biocides used on each tested species

**Species**	**Toxicant**	**EC_{50}**
P. tricornutum	$K_2Cr_2O_7$	29.21 mg/L [26.48; 32,35]
D. tertiolecta	$K_2Cr_2O_7$	89.12 mg/L [87.20; 91.45]
A. tonsa	Ni^{2+}	0.234 mg/L [0.184; 0.303]
F. enigmaticus	Cu^{2+}	75.5 mg/L [65.4; 87.8]
A. fischeri	Zn^{2+}	8.486 mg/L [7.575; 9,505]
